# Temporal Trends in Lung Cancer and Histological Subtype Incidence and Bayesian Projections to 2030 in China: Population-Based Quantitative Study

**DOI:** 10.2196/98324

**Published:** 2026-10-06

**Authors:** Xing Su, Jing Li, Xiaowei Hu

**Affiliations:** 1 Xihu District Center for Disease Control and Prevention Hangzhou, Zhejiang China

**Keywords:** lung cancer, trend, histological subtype, prediction, age-period-cohort

## Abstract

**Background:**

Lung cancer is the leading cause of cancer-related death globally, with a significant burden in China, which accounts for more than one-third of the world’s new cases and 40% of related mortalities. Despite evidence that low-dose computed tomography screening can reduce mortality, its limited uptake in China means that most patients are diagnosed at an advanced stage, contributing to a low 5-year survival rate compared to high-income countries. Although overall incidence trends have been well documented, there is a scarcity of population-based data on the temporal trends of different histological subtypes of lung cancer in China.

**Objective:**

This study aimed to analyze population-based cancer registry data to assess lung cancer incidence trends by histological subtype and sex from 2001 to 2020 and to forecast future incidence rates through 2030 in Xihu District, Hangzhou, Zhejiang Province, eastern China.

**Methods:**

The study used population-based surveillance data from the Xihu Cancer Registry from 2001 to 2020. Statistical analyses were conducted to assess the burden of each tumor subtype, including the average annual percentage change (AAPC) to evaluate temporal trends and the Bayesian age-period-cohort (BAPC) model to forecast incidence rates through 2030.

**Results:**

Lung cancer incidence rates were generally low among individuals aged <45 years but escalated markedly with age, reaching a peak of 567.6 per 100,000 person-years in among those aged 80 to 84 years. Notably, a significant rise in incidence was observed among those aged 30 to 44 years in recent years. Sex-based differences in histological distribution were pronounced. Adenocarcinoma accounted for 66.65% (2322/3484) of female cases compared to 37.1% (1795/4838) of male cases, while squamous-cell carcinoma was substantially less common in female cases (86/3484, 2.47% compared to 829/4838, 17.13% in male cases). Among male cases, the age-standardized incidence rates (ASIRs) of adenocarcinoma increased from 1.39 to 42.18 per 100,000 between 2001 and 2020; among female cases, the corresponding increase was even more pronounced, rising from 2.81 to 67.37 per 100,000. Joinpoint regression analysis demonstrated a consistent long-term increase in adenocarcinoma (AAPC=35.1, 95% CI 14.2-50.2), with peak acceleration observed from 2005 to 2012 (annual percentage change [APC]=76.7, 95% CI 45.2-124.2), and a steady increase in large-cell and other specified carcinomas (AAPC=32.7, 95% CI 12.5-45.7), while unspecified carcinoma showed a long-term decline (AAPC=−3.1, 95% CI −14.2 to −1.1). BAPC projections indicated that adenocarcinoma ASIR will continue to surge between 2020 and 2030, emerging as the dominant histological subtype, while unspecified carcinoma, squamous-cell carcinoma, and small-cell lung cancer are anticipated to decline modestly.

**Conclusions:**

The increase in lung cancer incidence, particularly among female participants, underscores the need for effective prevention and control strategies, including targeted interventions to reduce smoking.

## Introduction

Globally, lung cancer represents one of the most commonly diagnosed malignancies and remains the leading cause of cancer-related mortality. According to the latest GLOBOCAN estimates, approximately 2.5 million new lung cancer cases and 1.8 million deaths were recorded in 2022 worldwide [1]. China is one of the countries most affected by lung cancer. According to the latest national cancer statistics, lung cancer is the leading cause of both cancer incidence and mortality in China, with approximately 1,061,000 new cases and 733,000 deaths recorded in 2022, accounting for approximately 43% of global lung cancer incidence and 40% of global lung cancer mortality [2]. Cancer incidence rates often differ markedly across regions and between sexes, influenced by variations in lifestyle and environmental factors. In urban settings, the number of incident cases reached 457,000, representing a 1.4-fold increase compared to rural regions, with higher incidence observed among male patients than female patients [3]. Zhang et al [4] indicated a modest rise in the age-standardized incidence rate (ASIR) for men (annual percentage change [APC] 0.4%) alongside a decline for women (APC −0.2%) over the period from 2000 to 2014. In contrast, death rates increased during more recent periods, rising from 5.47 per 100,000 in the mid-1970s to 17.27 per 100,000 in the early 1990s and further to 30.83 per 100,000 during the 2000s [5].

Many studies have confirmed the mortality-reducing benefits of low-dose computed tomography (LDCT) for lung cancer screening [6,7]. However, lung cancer screening has not been widely adopted in China. Consequently, most cases are detected at advanced stages, leaving only a small proportion diagnosed early, when treatment is most effective [8]. The high proportion of late-stage diagnoses is reflected in the low 5-year survival rate of lung cancer in China [9], reflecting the continued predominance of late-stage diagnoses at presentation [10]. This rate was substantially lower than the 5-year survival rates reported in other high-income countries, which ranged from approximately 22% in Australia to 32% in Japan [11]. Besides stage and anatomical location, cancer histology (eg, adenocarcinoma, squamous-cell carcinoma, and large-cell carcinoma) is another major determinant of prognosis and treatment approach [12].

Although temporal patterns of lung cancer incidence have been extensively studied [13-15], data on histological subtype trends in China remain limited. One previous study reported trends in lung cancer incidence rates by histological type in Sichuan, China [16]; however, this study relied on data from a large hospital rather than population-based surveillance data. Population-based surveillance data can provide a more representative sample of the population and help identify trends and patterns in the incidence and prevalence of different subtypes of lung cancer. Therefore, this study aimed to perform a population-based analysis using cancer registry data to assess lung cancer trends, focusing on the most prevalent histological types from 2001 to 2020. Additionally, we projected future incidence rates (2020-2030) across different lung cancer subtypes.

## Methods

### Data Source

This study examined incident lung cancer cases in Xihu District, Zhejiang, China, from 2001 to 2020. Xihu District is 1 of the 11 districts of Hangzhou, located in Zhejiang Province, eastern China. It has an area of 7265 km^2^ with about 1 million inhabitants and is situated between 29°16′N and 30°03′N and 118°20′E and 119°15′E. The Xihu Cancer Registry was founded in 1986 and is a member of the International Association of Cancer Registries. Incident cancer cases were classified using the *International Classification of Diseases for Oncology, 3rd Edition* (*ICD-O-3*) [17] and the *International Statistical Classification of Diseases, 10th Revision* (*ICD-10*) [18]. Lung cancer, categorized by *ICD-10* codes C34.0-C34.9, was identified and classified. Data were extracted from the Cancer Registration Database, with preliminary reviews conducted by local cancer registries. Case reporting primarily relied on hospital records, community health centers, and village-level health care providers. Additional sources included the national health insurance system, the rural cooperative medical scheme, and vital statistics. Detailed methodologies for data collection, management, and analysis in China’s cancer registries have been described elsewhere [2]. Population data for the study period were obtained from the Xihu Bureau of Statistics. The registered population of the district was approximately 510,000 persons in 2001 and 989,000 persons in 2020, with a mean annual population of approximately 760,000 persons over the study period. This research followed the Guidelines for Accurate and Transparent Health Estimates Reporting (GATHER) [19].

### Ethical Considerations

This study was approved by the ethics committee of Xihu District Center for Disease Control and Prevention (2023-065). As this study used retrospective, population-based cancer registry data from the Xihu Cancer Registry, the requirement for informed consent from individual participants was waived by the ethics committee, given the observational and noninterventional nature of the study. All data used in this study were fully anonymized and deidentified prior to analysis, ensuring the privacy and confidentiality of all individuals. No participant compensation was applicable, as this study involved a secondary analysis of existing registry data.

### Histological Classification

All cases were histologically subtyped according to the most recent World Health Organization (WHO) classification system for lung tumors. We divided lung cancer histology, coded as *ICD-O-3*, into the following subtypes: small-cell lung cancer (SCLC; *ICD-O-3* 8002 and 8041-8045), adenocarcinoma (*ICD-O-3* 8015, 8050, 8140-8141, 8143-8145, 8147, 8190, 8201, 8211, 8250-8255, 8260, 8290, 8310, 8320, 8323, 8333, 8401, 8440, 8470-8471, 8480-8481, 8490, 8503, 8507, 8550, 8570-8572, and 8574, 8576), squamous-cell carcinoma (*ICD-O-3* 8051-8052, 8070-8076, 8078, 8083-8084, 8090, 8094, 8120, and 8123), large-cell and other specified carcinomas (8003-8004, 8012-8014, 8021-8022, 8030-8035, 8082, 8200, 8240-8241, 8243-8246, 8249, 8430, 8525, 8560, 8562, and 8575), and unspecified carcinoma (8000-8001, 8010-8020, 8046, 8230) [20].

### Statistical Analysis

To evaluate the disease burden across different tumor subtypes, we analyzed both crude incidence rates and ASIRs. The ASIRs were calculated using the World Standard Population, following the Segi methodology [21]. For longitudinal trend analysis across the study period (2001-2020), we used the average annual percentage change (AAPC) to quantify temporal variations in age-standardized lung cancer incidence rates. Joinpoint regression was used to estimate the AAPC, with a maximum of 2 joinpoints allowed the ASIR to balance model complexity and avoid overfitting given the 20 annual data points [22]. We modeled temporal trends by fitting a linear regression to log-transformed rates (ln(ASIR)=Y=α+βX+ε), where β quantifies the direction and magnitude of annual change. The AAPC was then calculated as 100×(e^β^−1). Instead of stratifying by gender, we incorporated sex as a covariate in the joinpoint model to explicitly test for differential trends between male patients and female patients. If the interaction between sex and year was significant (*P*<.05), we reported sex-stratified AAPCs; otherwise, we reported the overall trend adjusted for sex.

To forecast the incidence rates of lung cancer through 2030, we used a statistical model known as the Bayesian age-period-cohort (BAPC) model. Age-period-cohort modeling has become an established analytical framework for examining temporal patterns in cancer epidemiology, particularly for incidence and mortality trends. In this case, the incidence rates are examined in relation to 3 distinct time frames: age, period, and cohort. These time frames help account for various factors that influence the rates, such as consistent external factors linked to aging, external factors that affect all age groups equally during a specific period (eg, pollution or medical advancements), and generational effects (eg, malnutrition among children during or after wars). The projection of incidence rates using age-period-cohort models is particularly important due to changes in demographics, as well as advancements in therapeutic and diagnostic practices. BAPC models offer methodological advantages over conventional approaches by integrating prior knowledge about temporal smoothing across all 3 dimensions. This inclusion helps reduce random fluctuations and enhances the accuracy of the projections [23]. In the BAPC model, we established prior distributions for the effects of age, period, and cohort. These distributions smooth each data point based on the 2 preceding points, resembling a second-order random walk pattern. Trend analysis was conducted using Joinpoint software (version 4.8.0.1; Statistical Research and Applications Branch, National Cancer Institute), with complementary analyses performed in the R statistical environment (version 4.0.0; R Foundation for Statistical Computing).

## Results

Figure 1 illustrates the temporal trends in lung cancer incidence among different age groups from 2001 to 2020. The incidence rates of lung cancer were generally low among individuals aged <45 years. However, as age increased, the incidence rates of lung cancer also escalated, reaching their peak among those aged 80 to 84 years, with a maximum of 567.6 per 100,000 person-years recorded during the study period. Notably, we observed a significant rise in the incidence rates among younger individuals in recent years, particularly those aged 30 to 44 years. In Figure 2, we present the temporal trends in the ASIRs for lung cancer based on histological type. The ASIR for adenocarcinoma exhibited a consistent and substantial increase from 2001 to 2020. On the other hand, there were some variations in the ASIR for unspecified carcinoma during the same period for both sexes. Regarding other histological types, the ASIRs remained stable or slightly decreased between 2001 and 2020.

**Figure 1:**
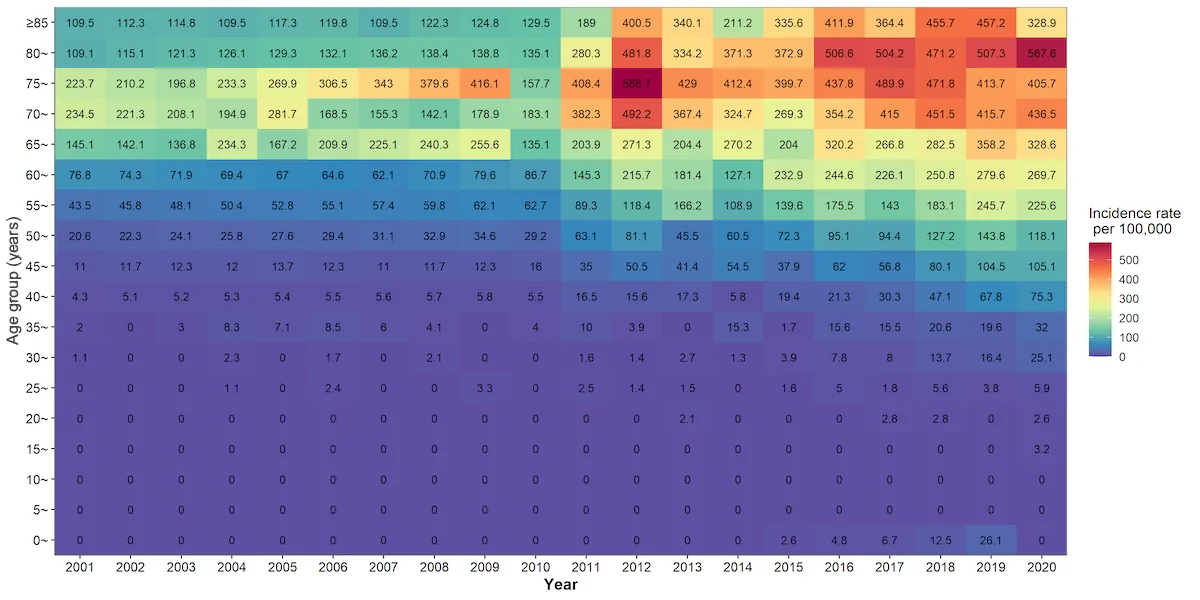
Temporal trends in lung cancer incidence rate by age groups (2001-2020).

**Figure 2:**
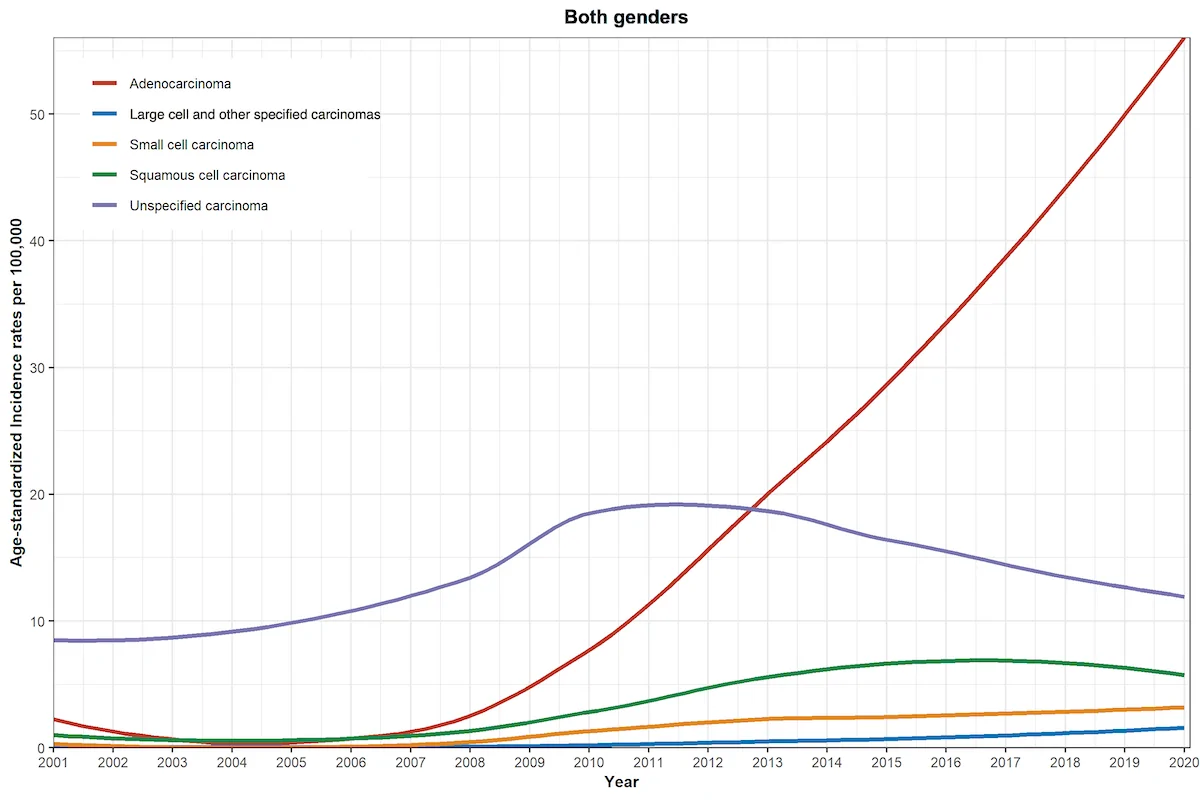
Temporal trends in age-standardized incidence rates for lung cancer based on histological subtypes.

Table 1 illustrates the relative change in ASIR of lung cancer between 2001 and 2020, categorized by histological type and age. Notably, in male patients, with the exception of unspecified carcinoma, all other histological types experienced an increase in ASIR over the mentioned time period. Of particular interest is the substantial rise in ASIR for adenocarcinoma, which escalated from 1.39 per 100,000 in 2001 to 42.18 per 100,000 in 2020. Similarly, among female patients, the highest increase in ASIR was also observed in adenocarcinoma, surging from 2.81 per 100,000 in 2001 to 67.37 per 100,000 in 2020. In contrast to the male population, we noted a rise in ASIR for squamous-cell carcinoma among the female population, with rates increasing from 0.35 per 100,000 in 2001 to 0.50 per 100,000 in 2020.

**Table 1:** Estimated age-standardized incidence rates for lung cancer by histological subtypes and their percentage change in male and female participants.

Characteristics		Age-standardized incidence rates per 100,000 (95% CI)			Percentage change (%)
		2001	2020		
**Male**					
	Small-cell carcinoma	0.42 (0.33-2.78)	4.84 (3.17-7.72)	1052.38	
	Squamous-cell carcinoma	1.04 (0.73-4.21)	9.51 (7.01-13.15)	814.42	
	Large-cell and other specified carcinomas	0.21 (0.02-2.83)	1.84 (0.87-4.19)	776.19	
	Adenocarcinoma	1.39 (0.94-4.32)	42.18 (36.61-48.77)	2934.53	
	Unspecified carcinoma	19.32 (16.28-25.34)	13.49 (10.3-17.44)	−30.18	
**Female**					
	Small-cell carcinoma	0 (0)	1.06 (0.38-3.25)	—^a^	
	Squamous-cell carcinoma	0.35 (0.17-1.44)	0.50 (0.10-2.58)	42.86	
	Large-cell and other specified carcinomas	0 (0)	1.34 (0.57-3.57)	—	
	Adenocarcinoma	2.81 (1.05-5.42)	67.37 (60.33-75.38)	2297.51	
	Unspecified carcinoma	12.34 (9.13-15.52)	10.19 (7.71-13.81)	−17.42	

^a^Not applicable.

Table 2 summarizes the demographic characteristics of the study population by sex, histology, age, diagnosis period, and stage. Histological distribution patterns differed markedly between sexes: unspecified carcinoma predominated in male participants (1832/4838, 37.87%), followed by adenocarcinoma (1795/4838, 37.10%) and squamous-cell carcinoma (829/4838, 17.13%), while adenocarcinoma accounted for two-thirds of female cases (2322/3484, 66.65%), with unspecified carcinoma (972/3484, 27.90%) and squamous-cell carcinoma (86/3484, 2.47%) being less common. Large-cell and other specified carcinomas represented the rarest subtype in both groups. Notable sex-based differences emerged, with female participants showing a 1.8-fold higher adenocarcinoma prevalence (2322/3484, 66.65% vs 1795/4838, 37.10%) but an 86% lower squamous-cell carcinoma incidence (86/3484, 2.47% vs 829/4838, 17.13%) than male participants. Age-stratified analysis revealed that adenocarcinoma predominated in younger patients (≤40 years; 854/1077, 79.29%), while unspecified carcinoma was most prevalent in the oldest group (≥80 years; 716/1242, 57.65%).

**Table 2:** Distribution of patients included in the analyses by sex, age, and period of diagnosis according to histological subtype.

Characteristics			Small-cell carcinoma, n (%)		Squamous-cell carcinoma, n (%)		Large-cell and other specified carcinomas, n (%)		Adenocarcinoma, n (%)		Unspecified carcinoma, n (%)
**Age group (years)**											
	<40 (n=1077)	23 (2.14)		28 (2.6)		18 (1.67)		854 (79.29)		154 (14.3)	
	40-59 (n=2157)	75 (3.48)		139 (6.44)		22 (1.02)		1470 (68.15)		451 (20.91)	
	60-69 (n=2129)	115 (5.4)		234 (10.99)		39 (1.83)		1230 (57.77)		511 (23.99)	
	70-79 (n=2030)	93 (4.58)		288 (14.19)		28 (1.38)		882 (43.45)		739 (36.4)	
	≥80 (n=1242)	40 (3.22)		140 (11.27)		18 (1.45)		328 (26.41)		716 (57.65)	
**Sex**											
	Male (n=4838)	298 (6.16)		829 (17.13)		84 (1.74)		1795 (37.1)		1832 (37.87)	
	Female (n=3484)	71 (2.04)		86 (2.47)		33 (0.95)		2322 (66.65)		972 (27.9)	
**Period of diagnosis**											
	2001-2005 (n=1357)	60 (4.42)		166 (12.23)		21 (1.55)		521 (38.39)		589 (43.4)	
	2006-2010 (n=1598)	76 (4.76)		186 (11.64)		17 (1.06)		645 (40.36)		674 (42.18)	
	2011-2015 (n=1963)	96 (4.89)		230 (11.72)		19 (0.97)		842 (42.92)		776 (39.55)	
	2016-2020 (n=3404)	137 (3.98)		333 (9.68)		60 (1.74)		2109 (61.29)		765 (22.23)	

Table 3 presents the results of a joinpoint regression analysis of lung cancer ASIR by histological type from 2001 to 2020. The interaction between sex and year was not significant (*P*>.05); therefore, we reported the overall trend adjusted for sex. In terms of specific histology, we observed a consistent long-term decrease in unspecified carcinoma (AAPC=−3.1, 95% CI −14.2 to −1.1) and a steady long-term increase in large-cell and other specified carcinomas (AAPC=32.7, 95% CI 12.5-45.7). The greatest increase in ASIR was observed for adenocarcinoma (AAPC=35.1, 95% CI 14.2-50.2) between 2001 and 2020, with peak acceleration observed from 2005 to 2012 (APC=76.7, 95% CI 45.2-124.2).

Figure 3 displays the observed and predicted trends in the ASIR of lung cancer by histological subtype from 2001 to 2030. Our analysis indicates that the ASIR of adenocarcinoma is projected to undergo a substantial surge between 2020 and 2030, emerging as the dominant histological subtype among the different histological subtypes. Conversely, the ASIRs of other histological subtypes of lung cancer, namely unspecified carcinoma, squamous-cell carcinoma, and SCLC, are anticipated to experience modest declines until 2030. The ASIR of large-cell and other specified carcinomas is expected to persist at a consistently low level.

**Table 3:** Joinpoint regression analysis of lung cancer incidence rates by histological subtype (2001-2020).

Histological subtypes	The whole study period			Period 1			Period 2			Period 3	
	Years	AAPC^a^ (95% CI)	Years		APC^b^ (95% CI)	Years		APC (95% CI)	Years		APC (95% CI)
Overall	2001-2020	13.1 (6.8 to 18.3)	2001-2005		4.2 (1.3 to 6.9)	2005-2012		26.9 (2.3 to 67.1)	2012-2020		5.9 (2.8 to 9.1)
Small-cell carcinoma	2001-2020	19.2 (6.3 to 27.1)	2001-2005		10.1 (7.2 to 15.4)	2005-2012		76.3 (8.3 to 123.1)	2012-2020		2.4 (−3.8 to 9.0)
Squamous-cell carcinoma	2001-2020	12.2 (1.6 to 28.9)	2001-2005		6.7 (−2.1 to 11.9)	2005-2012		60.0 (−2.1 to 178.1)	2012-2020		0.7 (−6.1 to 7.9)
Large-cell and other specified carcinomas	2001-2020	32.7 (12.5 to 45.7)	2010-2012		3.2 (−4.1 to 16.3)	2012-2020		20.7 (7.0 to 25.5)	—^c^		—
Adenocarcinoma	2001-2020	35.1 (14.2 to 50.2)	2001-2005		12.3 (4.2 to 25.1)	2005-2012		76.7 (45.2 to 124.2)	2012-2020		7.3 (−3.2 to 18.2)
Unspecified carcinoma	2001-2020	−3.1 (−14.2 to −1.1)	2001-2020		−3.1 (−14.2 to −1.1)	—		—	—		—

^a^AAPC: average annual percentage change.

^b^APC: annual percentage change.

^c^Not applicable.

**Figure 3:**
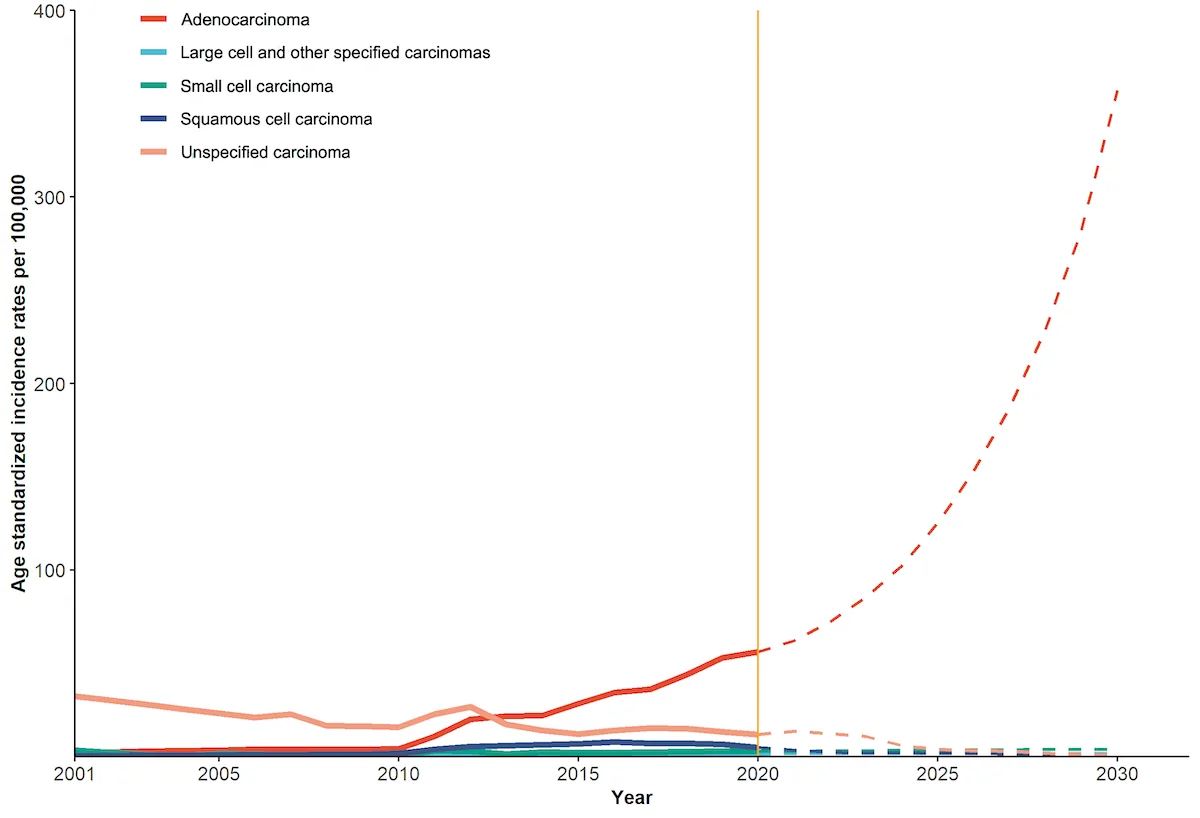
Observed and predicted trends in age-standardized incidence rates for lung cancer by histological subtypes (2001-2030).

## Discussion

### Principal Findings

This study examined temporal patterns and projected future trends in lung cancer incidence in Xihu, Zhejiang, over the coming decade. Our findings revealed a marked rise in lung cancer rates, aligning with national trends observed across China. Histologically, adenocarcinoma incidence showed sustained growth, while small-cell and squamous-cell carcinomas exhibited stable trends. Notably, female lung cancer cases increased significantly, mirroring epidemiological patterns reported internationally. Together, these findings speak directly to the aims of this study. They delineate 2 decades of histology-specific incidence trends from population-based registry data and indicate that the rising burden, driven predominantly by adenocarcinoma, is likely to persist through 2030.

In line with other studies [24], our findings indicate that the number of lung cancer diagnoses and the ASIR increased during the period from 2001 to 2020. This upward trend may be attributed to factors such as tobacco use, population aging, and advancements in lung cancer detection and treatment in China. Tobacco use stands out as a major risk factor that contributes significantly to the escalating lung cancer crisis [3]. In response to this public health challenge, China’s national “Healthy China 2030” initiative has established ambitious tobacco control targets, including reducing smoking prevalence to <20% by the target year [25]. Various measures have been implemented to achieve this goal, including increasing the cost and taxes of tobacco products and establishing smoke-free zones. Consequently, the smoking rate has shown a substantial decline from 30.2% to 26.9% between 2000 and 2015, as reported by the WHO. Paradoxically, although smoking prevalence has demonstrably declined, China’s total number of smokers grew by approximately 5 million (from 310 to 315 million) during the study period [26]. Therefore, considering population growth and population aging, additional efforts are necessary to reduce the incidence of lung cancer by curbing the smoking rate. Furthermore, the growing proportion of older individuals in China’s population also contributes to the rising incidence of lung cancer. Presently, China faces a significant aging challenge, with approximately 11.5% of the total population aged >65 years in 2019 [27]. Consequently, it is crucial to focus on monitoring and addressing the incidence and mortality of lung cancer in the middle-aged and elderly population.

For histology-specific lung cancer trends, we observed a consistent rise in adenocarcinoma over a long period, while SCLC and squamous-cell carcinoma reached a plateau. Despite adenocarcinoma being the most prevalent subtype for both men and women, women exhibited an even greater proportion of this cell type. Moreover, women are more likely to develop adenocarcinoma in situ, which is an early-stage, noninvasive abnormal growth [28]. Our results align with previous studies on changing histology patterns of lung cancer in Osaka (Japan) [29], South Korea [30], and other countries [15]. Notably, stabilization of adenocarcinoma rates among post-1950 birth cohorts has been reported in Canada, Denmark, and Australia [15]. The specific risk factors responsible for the rising incidence of adenocarcinoma remain uncertain. One possible explanation for the increasing rates could be the growing exposure to secondhand smoke [31]. It is believed that sidestream smoke, which is emitted from the burning end of a cigarette, may carry lung carcinogens that are unique to tobacco and deposit them in the outer areas of the lungs. Moreover, previous studies have established associations between airborne pollutants (including particulate matter with a diameter of ≤2.5 μm and nitrogen oxides) and increased susceptibility to lung adenocarcinoma development [32]. Furthermore, improvements in diagnostic pathology, including the widespread adoption of immunohistochemistry and the updated WHO classification of lung tumors, may have contributed to the observed rise in adenocarcinoma, as cases previously classified as unspecified carcinoma (AAPC=−3.1) were more precisely reclassified over time.

Global epidemiological data reveal an increasing trend in female lung cancer incidence, with particularly notable rises observed across North American and European populations in recent decades. The ratio of men to women affected by lung cancer is gradually decreasing [1]. Our findings align with the global trend, indicating a significant increase in lung cancer cases among women. Although smoking prevalence among women remains comparatively low, their lung cancer incidence is higher than that observed in several European nations. This discrepancy may stem from exposure to indoor air pollutants, including emissions from inefficient coal stoves and cooking oil vapors in poorly ventilated spaces [33]. Secondhand smoke exposure represents another significant risk factor, disproportionately affecting Chinese women [34], potentially accounting for the elevated female lung cancer burden identified in our analysis. Biological factors may further contribute to this pattern, as evidenced by the retrospective findings of Kligerman and White [28], which suggest that women demonstrate heightened sensitivity to tobacco and environmental carcinogens, potentially mediated through increased CYP1A1 expression and p53 mutations. Additionally, estrogen has been shown to promote lung cancer progression via interactions with both estrogen receptors and epidermal growth factor receptor pathways, suggesting potential therapeutic targets for intervention [35].

The epidemiological patterns identified in this study have direct implications for lung cancer screening and prevention strategies. The sustained rise in adenocarcinoma, particularly among women with low smoking prevalence, highlights a critical limitation of current LDCT screening guidelines, which remain primarily focused on heavy smokers. We therefore advocate for the expansion of screening eligibility to encompass individuals with significant indoor air pollution exposure, such as cooking oil fumes and coal combustion emissions, as well as those with a family history of lung cancer. On the prevention side, a differentiated, subtype-specific approach is warranted: tobacco control efforts should remain a priority to sustain the observed plateau in squamous-cell and small-cell carcinoma incidence, while nontobacco-focused interventions, including improved indoor ventilation, clean cooking technologies, and stricter ambient air quality regulations, are essential to address the continuing rise in adenocarcinoma, particularly among women.

On the basis of our prediction analysis, we anticipate a continued rise in lung cancer rates, specifically for adenocarcinoma. The prevalence of smoking among Chinese men has remained consistently high since the 1980s. Although male smoking rates peaked in 1990 and have somewhat decreased since then [36], if these trends persist, we can expect further reductions in the incidence of lung cancer subtypes strongly linked to smoking, such as small-cell, squamous-cell, and large-cell carcinomas. However, the incidence of adenocarcinomas may continue to increase, possibly in proportion to the overall rise in lung cancer cases. To gain a better understanding of current trends, particularly in nonsmokers, it is essential to conduct additional studies that explore variations in the response to smoking exposure based on histology and investigate the associations between traditional risk factors for lung cancer and other factors with specific histological types.

Before interpreting our findings, it is important to acknowledge several potential limitations. First, our research is a descriptive study that relies on cancer registry data, which means that we cannot establish causal relationships to explain the temporal trends of lung cancer. The Xihu Cancer Registry follows standard quality control procedures, including case verification, data completeness checks, and regular audits; however, variations in diagnostic practices and registration protocols over the 20-year study period may have introduced some degree of inconsistency into the data. Second, there is a possibility of underreporting and missed diagnoses, which could introduce bias into the cancer registration data and result in an underestimation of lung cancer cases. Finally, our prediction model assumes that current and past trends will continue unchanged, disregarding any potential advancements in the prevention or diagnosis of lung cancer that could affect future incidence rates. Furthermore, as a statistical model, the BAPC projections cannot incorporate the potential impact of emerging public health interventions, which may cause actual future incidence rates to deviate from the predicted trajectory. Therefore, it would be advisable to conduct future studies that incorporate these influential factors to develop more comprehensive forecast models.

### Conclusions

Drawing on 2 decades of population-based registry data, this study shows that the lung cancer burden in Xihu is not only growing but also changing in character, with adenocarcinoma, particularly among women with low smoking prevalence, accounting for an increasing share of the disease. The significance of these findings extends beyond a single district. They suggest that lung cancer control in China can no longer rely on tobacco control and smoker-targeted screening alone: screening frameworks will need to evolve toward risk-adapted criteria that incorporate nontobacco exposures such as indoor air pollution and family history, and prevention strategies will need to become subtype-aware. More broadly, as many low- and middle-income countries undergo similar epidemiological transitions, population-based cancer surveillance capable of resolving histological subtypes will be indispensable for anticipating, rather than merely reacting to, the changing epidemiology of lung cancer, and etiological research into nonsmoking risk factors should be prioritized to inform the next generation of prevention policies.

## Abbreviations

AAPC: average annual percentage change
APC: annual percentage change
ASIR: age-standardized incidence rate
BAPC: Bayesian age-period-cohort
GATHER: Guidelines for Accurate and Transparent Health Estimates Reporting
ICD-10: International Statistical Classification of Diseases, 10th Revision
ICD-O-3: International Classification of Diseases for Oncology, 3rd Edition
LDCT: low-dose computed tomography
SCLC: small-cell lung cancer
WHO: World Health Organization

## Data Availability

The datasets generated and analyzed during the current study are available from the corresponding author on reasonable request.
